# Methylglyoxal-Dependent Glycative Stress and Deregulation of SIRT1 Functional Network in the Ovary of PCOS Mice

**DOI:** 10.3390/cells9010209

**Published:** 2020-01-14

**Authors:** Giovanna Di Emidio, Martina Placidi, Francesco Rea, Giulia Rossi, Stefano Falone, Loredana Cristiano, Stefania Nottola, Anna Maria D’Alessandro, Fernanda Amicarelli, Maria Grazia Palmerini, Carla Tatone

**Affiliations:** 1 Department of Life, Health and Environmental Sciences, University of L’Aquila, 67100 L’Aquila, Italy; giovanna.diemidio@univaq.it (G.D.E.); martina.placidi@graduate.univaq.it (M.P.); frea@unite.it (F.R.); grossi@unite.it (G.R.); stefano.falone@univaq.it (S.F.); loredana.cristiano@univaq.it (L.C.); annamaria.dalessandro@univaq.it (A.M.D.); fernanda.amicarelli@univaq.it (F.A.); mariagrazia.palmerini@univaq.it (M.G.P.); 2 Department of Anatomy, Histology, Forensic Medicine and Orthopaedics, 00161 Roma, Italy; stefania.nottola@uniroma1.it

**Keywords:** PCOS, advanced glycation end-products, methylglyoxal, glycative stress, glyoxalases, SIRT1, SIRT3, oocyte quality, mitochondria, oxidative stress

## Abstract

Advanced glycation end-products (AGEs) are involved in the pathogenesis and consequences of polycystic ovary syndrome (PCOS), a complex metabolic disorder associated with female infertility. The most powerful AGE precursor is methylglyoxal (MG), a byproduct of glycolysis, that is detoxified by the glyoxalase system. By using a PCOS mouse model induced by administration of dehydroepiandrosterone (DHEA), we investigated whether MG-dependent glycative stress contributes to ovarian PCOS phenotype and explored changes in the Sirtuin 1 (SIRT1) functional network regulating mitochondrial functions and cell survival. In addition to anovulation and reduced oocyte quality, DHEA ovaries revealed altered collagen deposition, increased vascularization, lipid droplets accumulation and altered steroidogenesis. Here we observed increased intraovarian MG-AGE levels in association with enhanced expression of receptor for AGEs (RAGEs) and deregulation of the glyoxalase system, hallmarks of glycative stress. Moreover, DHEA mice exhibited enhanced ovarian expression of SIRT1 along with increased protein levels of SIRT3 and superoxide dismutase 2 (SOD2), and decreased peroxisome proliferator-activated receptor gamma co-activator 1 alpha (PGC1α), mitochondrial transcriptional factor A (mtTFA) and translocase of outer mitochondrial membrane 20 (TOMM20). Finally, the presence of autophagy protein markers and increased AMP-activated protein kinase (AMPK) suggested the involvement of SIRT1/AMPK axis in autophagy activation. Overall, present findings demonstrate that MG-dependent glycative stress is involved in ovarian dysfunctions associated to PCOS and support the hypothesis of a SIRT1-dependent adaptive response.

## 1. Introduction

Polycystic ovarian syndrome (PCOS) is a common and complex endocrine disorder affecting 4–21% of women in reproductive age [1,2]. According to the Rotterdam criteria, the PCOS classic phenotype consists of hyperandrogenism, oligo-ovulation and polycystic ovaries, in association with insulin resistance, metabolic disorders and infertility [3]. Long-term risks conferred by PCOS include diabetes and cardiovascular disease. Although underlying mechanisms have not been fully elucidated yet, a crucial role in the pathogenesis of PCOS is played by oxidative stress [4,5].

In the last decade, new players have been implicated in the pathogenesis of PCOS, the advanced glycation end products (AGEs). Glycation is a spontaneous non-enzymatic reaction of reducing sugars with free amino groups of proteins, DNA and lipids that forms Amadori products. The Amadori products undergo a variety of irreversible dehydration and rearrangement reactions that ultimately lead to the formation of AGEs [6]. Physiological glycation involves the modification of proteins by reactive α-oxoaldehydes—particularly glyoxal, methylglyoxal and 3-deoxyglucosone. The most powerful precursor of AGEs is methylglyoxal (MG), a low-molecular weight dicarbonyl compound derived from metabolic processes [7]. MG reacts primarily with arginine residues to form hydroimidazolones and argpyrimidine [8,9], here referred to as MG-AGEs. Intracellular MG detoxification relies on the activity of the so-called glyoxalase system, composed of glyoxalase 1 (GLO1) and glyoxalase 2 (GLO2). GLO1 converts MG to S-d-lactoylglutathione in a glutathione (GSH)-dependent manner, whereas GLO2 catalyses the hydrolysis of S-d-lactoylglutathione to d-lactate, thus regenerating GSH [10]. MG build-up may derive from increased glycolytic metabolism and/or reduced glyoxalase activity, which may occur as a result of reduced GSH availability (e.g., oxidative stress) and/or decreased glyoxalase expression (e.g., aging) [10]. The accumulation of MG affects mitochondrial proteins and increases AGEs, which can also activate receptor-mediated pro-oxidant signalling pathways, thus generating a vicious cycle. Therefore, oxidative stress is considered an important aspect of the glycative burden, here referred to as glycative stress [10].

Increased levels of AGEs have been detected in the serum and ovary of women affected by PCOS [11,12,13]. Moreover, serum AGEs correlate positively with altered glucose metabolism, age and factors related to obesity, dyslipidaemia, hyperglycaemia and insulin resistance in patients undergoing in vitro fertilization (IVF) [14]. Further evidence for the role of AGEs in PCOS was provided by measurements in granulosa cells and serum of soluble receptors for AGEs (s-RAGE), a circulating isoform of RAGE that can neutralize the ligand-mediated damage [15]. A potential role of MG in PCOS was supported by the finding that dietary glycotoxins and hyperandrogenic status decrease GLO1 activity in rat ovaries, possibly contributing to increased AGE accumulation in granulosa cells [16]. We have recently shown in mice that dietary MG increased serum level of androstenedione, which may suggest a role of MG and AGEs in the hyperandrogenic status associated with PCOS. Moreover, we observed in the same model that the response to MG-dependent glycative stress in the female gonad involved a Sirtuin 1 (SIRT1) functional network [17]. SIRT1, a NAD^+^-dependent enzyme with deac(et)ylase activity, is the most studied member of the sirtuin family playing a key role during folliculogenesis by regulating mitochondrial biogenesis, oxidative stress defense and energy homoeostasis [18,19].

In the present work, we hypothesized that MG-dependent glycative stress participates in ovarian PCOS phenotype and explored whether this condition is associated with deregulation of SIRT1 functional network regulating mitochondrial physiology and cell survival. To this end, we relied on a dehydroepiandrosterone (DHEA)-induced PCOS model developed in CD1 mice [20], known to share many of the salient features with human PCOS patients [21]. The occurrence of PCOS was confirmed by analysing different parameters including follicle population, ovarian fibrosis, dyslipidemia, oestrous cycle, ovulation, oocyte quality and oxidative stress. Glycative stress was assessed by evaluating ovarian accumulation of MG-AGEs, expression of RAGEs and glyoxalases. In particular, we focused on argpyrimidine, an MG-AGE, which has known to be correlated with mouse and human ovarian dysfunctions [22,23,24]. Among the proteins that mediate the SIRT1-dependent action on mitochondria, a key role is played by peroxisome proliferator-activated receptor gamma co-activator 1 alpha (PGC1α) [25], the superoxide dismutase 2 (SOD2) and SIRT3, along with mitochondrial proteins such as mitochondrial transcriptional factor A (mtTFA), which is a downstream effector of SIRT1/PGC1α and indirect marker of mitochondrial number [25,26]. Under stress conditions, SIRT1 cooperates with AMP-activated protein kinase (AMPK) in order to restore energy balance or promoting cell death [27]. Thus, we explored whether in PCOS mouse ovaries activation of a SIRT1/AMPK axis may be associated with increased autophagy, a type of cell death recently found in PCOS ovarian cells [28,29].

Here we demonstrated that MG-dependent glycative stress is involved in ovarian dysfunctions associated to PCOS and suggest that this condition contributes to deregulation of SIRT1 functional network, mitochondrial mass and redox milieau and autophagy.

## 2. Materials and Methods

### 2.1. Animals

Outbred CD-1 mice (Charles River Italia s.r.l., Calco, Italy) were maintained in a temperature-controlled environment under a 12 h light/dark cycle (07:00–19:00) and free access to feed and water ad libitum. All the experiments were carried out in in conformity with national and international laws and policies (European Economic Community Council Directive 86/609, OJ 358, 1 Dec 12, 1987; Italian Legislative Decree 116/92, Gazzetta Ufficiale della Repubblica Italiana n. 40, Feb 18, 1992; National Institutes of Health Guide for the Care and Use of Laboratory Animals, NIH publication no. 85-23, 1985). The project was approved by the Italian Ministry of Health and the internal Committee of the University of L’Aquila.

Twenty 4 week-old, body weight 20–21 g, young CD-1 female mice were randomly assigned to two groups (10 for each): mice daily injected (sub cutaneously) with DHEA (6 mg/100 g body weight, 100 μL/mouse in sesame oil with 10% of 95% ethanol, Sigma-Aldrich, St. Louis, CO, USA) for 20 consecutive days (DHEA mice) [20,21]. The vehicle control group was injected with 0.09 mL sesame oil and 0.01 mL 95% ethanol daily for 20 consecutive days (control mice). Mice were sacrificed by an inhalant overdose of carbon dioxide (CO_2_, 10–30%), followed by cervical dislocation. All efforts were made to minimize suffering.

### 2.2. Estrous Cycle Determination

Analysis of vaginal smears was performed daily from the 7th day after the first injection of DHEA or vehicle. The stages of the estrous cycle were determined daily based on direct examination (“wet smear”) technique [30]. Vaginal cells were collected via saline lavage and then observed under light microscope with a 10× objective. Predominant nucleated epithelial cells and some cornified epithelial cells indicated the proestrous stage; predominant cornified squamous epithelial cells indicated the estrous stage; both cornified squamous epithelial cells and leukocytes indicated the metaestrous stage; and predominant leukocytes indicated the diestrous stage.

### 2.3. Superovulation Induction and Oocyte Collection

In order to obtain mature oocytes, at 48 h after the last treatment, the remaining three mice per group were treated for the induction of super ovulation by intraperitoneal injection of 10 IU pregnant mare’s serum gonadotropin (PMSG) (Folligon; Intervet-International, Boxmeer, Holland), followed by 10 IU human chorionic gonadotropin (hCG) (Profasi HP 2000; Serono, Roma, Italy) 48 h apart. Fifteen hours after hCG, oviducts were removed and oocytes arrested at metaphase II (MII) stage were isolated after a brief exposure to 0.3 mg/mL hyaluronidase (Sigma-Aldrich).

### 2.4. H&E Staining and Ovarian Follicle Classification and Counting

Part of the ovaries was sliced in half. One half of each ovaries was fixed in 3.7% paraformaldehyde (PFA) in PBS (Bio-Optica, Milan, Italy) for 12–16h Haematoxylin and Eosin (H&E) staining, dehydrated in the ascending series of alcohol, clarified in xylene and embedded in paraffin blocks. Samples were cut with a microtome (Leica SMR2000, Wetzlar, Germany) and sliced into 6 µm serial sections. Sections were then deparaffined and hydrated through xylenes and descending series of alcohol, stained with H&E according to the manufacturer’s instruction (Bio Optica, Milan, Italy) and observed by light microscopy (Zeiss Axiostar Plus, Oberkochen, Germany). Follicle classification and counting was performed by counting at least three serial sections/slide (20 slices in average for ovary), spaced ~50 µm each.

Stained ovarian follicles were classified as normal or degenerated for the qualitative evaluation. The normal follicles were those that presented the complete basal membrane, absence of pyknotic bodies in the oocyte nucleus, without signs of oocyte and/or granular degeneration. Normal follicles were classified according Gougeon’s classification [31] in: (i) primordial follicle, oocyte surrounded by a single layer of flattened pre-granulosa cells; (ii) primary follicle, oocyte showing a single layer of cuboidal granulosa cells; (iii) secondary follicle, with at least two complete layers of granulosa cells; and (iv) antral follicle, with development of an antral cavity.

### 2.5. Heidenhain’s AZAN Trichrome Staining

Paraffin embedded sections of formalin-fixed ovarian tissue were deparaffinized and hydrated through xylenes and graded alcohol series and processed for trichrome staining (Electron Microscopy Sciences, Danvers, MA, USA), according to manufacturer’s instructions.

### 2.6. Ovarian Immunofluorescence Analysis

Some half of the ovaries were fixed in 3.7% PFA/PBS (Bio Optica, Milan, Italy) for immunofluorescence analysis or sunk in liquid nitrogen and stored at −80 °C until processing. The following immunofluorescence markers were used: 17 beta-hydroxysteroid dehydrogenase type 4 (17β-HSD4), translocase of outer mitochondrial membrane 20 (TOMM20), Von Willebrand Factor (vWF) and alpha smooth muscle actin (α-SMA). After fixation, samples were washed in PBS for 10 min at room temperature (RT) and then incubated with 3% BSA/PBS (Sigma-Aldrich) for 1 h at RT or avidin/biotin blocking followed by M.O.M mouse IgG blocking reagent (Vector Laboratories, Burlingame, CA, USA, when mouse antibodies were used), according to the manufacturer’s instructions. After washing with PBS, ovarian sections were incubated with the following primary antibodies: rabbit polyclonal to 17β-HSD4, TOMM20 (1:100 and 1:400 respectively) (ThermoFisher Scientific, Rockford, IL, USA), vWF (1:500) (Dako, Glostrup, Denmark), all diluted in 3% BSA/PBS, for 1 h at RT and mouse monoclonal to α-SMA, (1:500) (Abcam, Cambridge, UK), diluted in M.O.M (Mouse on Mouse) diluent for 30 min, according to the manufacturer’s instructions. After washing with PBS, rabbit antibodies were revealed by donkey anti rabbit IgG Alexa Fluor 633 conjugated secondary antibody (1:2000) (Molecular Probes, Thermofisher), whereas mouse antibodies were recognized by biotinylated anti-mouse IgG (1:300, Vector), revealed by Fluorescein Avidin DCS (1:100), according to the manufacturer’s instructions. For the BODIPY staining, ovarian sections were incubated with 4,4-difluoro-1,3,5,7,8-pentamethyl 4-bora-3a,4a-diaza-sindacene (1 μg/mL, BODIPY 493/503 Molecular Probes, Invitrogen, Carlsbad, CA, USA), for 10 min, at RT. Finally, sections were mounted with Vectashield Mounting Medium with 4′,6-diamidino-2-phenylindole (DAPI) (Vector Laboratories, Burlingame, CA, USA) and examined under a Leica TCS SP5 confocal microscope (Mannheim, Germany).

Negative controls were performed by omitting the primary antibodies and substituting them with PBS solution containing 3% BSA or M.O.M diluent alone.

The evaluation and automated scoring of immunofluorescence (IF) signals was performed by using Image J 1.44p software according to Jensen [32].

### 2.7. Ovarian Immunohistochemical Analysis

Paraffin embedded sections of formalin-fixed ovarian tissue were deparaffinized and hydrated through xylenes and graded alcohol series. To increase the immunoreactivity, the sections were boiled in 10 mM citrate buffer (pH, 6.1 Bio-Optica, Milan, Italy) in a microwave at 720 W (3 cycles/3 min each). The sections were then subjected to treatment for blocking endogenous peroxidase activity (Dako). After thorough washing, sections were incubated with M.O.M mouse IgG blocking reagent overnight at 4 °C (Vector Laboratories) according to the manufacturer’s protocol. Then sections were incubated with mouse monoclonal to methylglyoxal (MG)-AGE (Arg-Pyrimidine, AGE06B, BioLogo, 1:100) antibody or rabbit polyclonal to 4-HNE (4 Hydroxynonenal, ab46545, Abcam, 1:100) diluted in M.O.M diluent for 30 min, according to the Vector Laboratories instructions. MG-AGE and 4-HNE were revealed by biotinylated anti-mouse and anti-rabbit IgG, respectively, followed by streptavidin- horseradish peroxidase (HRP), 3,3-diaminobenzidine (DAB) substrate buffer and DAB (Dako kit), according to manufacturer’s instructions. Counterstaining was performed with hematoxylin (Bio-Optica). Negative controls were performed by omitting primary antibody and substituting it with M.O.M diluent alone. Finally, sections were dehydrated and mounted with Neomount (Merck, Darmstadt, Germany). They were observed and photographed under a Leitz Laborlux S microscope (Germany) equipped with an Olympus digital compact camera. The evaluation and automated scoring of immunohistochemistry (IHC) signals was performed by using Image J 1.44p software (IHC profiler plugin) according to Varghese et al. [33].

### 2.8. Western Blot Analysis

Part of the ovaries stored at −80 °C were processed for protein extraction. Ovarian tissues were homogenized in RIPA buffer by repeated freeze/thaw cycles in liquid nitrogen. After centrifugation (14,000 rpm for 90 min at 4 °C), the supernatants were collected for protein analysis. Protein concentration was determined by BCA protein assay kit (Pierce, Rockford, IL, USA). Protein samples were separated by SDS-PAGE and transferred to a polyvinylidene difluoride membrane (Sigma-Aldrich). Non-specific binding sites were blocked for 1 h at room temperature with 5% not fat dry milk (Bio-Rad Laboratories, Segrate, Italy) in Tris-buffered saline containing 0.05% Tween 20 (TBS-T). Membranes were incubated with polyclonal rabbit anti-SIRT1 antibody (Ab12193, Abcam; 1:700), anti-SIRT3 (Ab86671, Abcam, Cambridge, UK; 1:500), anti-SOD2 antibody (Ab86087, Abcam; 1:1000), anti-GLO1 antibody (MA1-13029, Thermo Fisher; 1:400), anti-GLO2 antibody (Ab154108, Abcam; 1:500), anti-PGC1α antibody (SC-13067, Santa Cruz Biotechnology Inc., 1:500), anti-RAGE (PA1-075, ThermoFisher Scientific, 1:750), mouse anti-Methylglyoxal (MG)-AGE (argpyrimidine) monoclonal antibody (AGE06B, BioLogo; 1:250), anti-mtTFA antibody (SC-166965, Santa Cruz, 1:250), anti-17β-HSD4 antibody (PA5-21522, ThermoFisher Scientific, 1:250); anti-AMPKα1 antibody (AB-84049, Immunological Sciences, Rome, Italy, 1:500), anti-phospho-AMPKα1 antibody (S487, ABP-0619, Immunological Sciences, 1:500), anti-microtubule-associated protein light chain 3 (LC3) antibody (AB-83557, Immunological Sciences, 1:500), anti-p62 (also known as sequestosome-1) antibody (AB-83779, Immunological Sciences, 1:500) or anti-glyceraldehyde-3-phosphate dehydrogenase (GAPDH) (TA802519, OriGene Technologies Inc, 1:750) overnight at 4 °C, followed by incubation with horseradish peroxidase (HRP) conjugated anti-rabbit (BA1054, Boster Biological Technology Co., Ltd., 1:3000) or anti-mouse secondary antibody (Ab6728, Abcam, 1:2000) for 1 h at room temperature. After washing, specific immunoreactive complexes were detected by ECL kit (Thermo Scientific, Waltham, MA, USA) and Uvitec Cambridge system (Alliance series, Cambridge, UK). The bands were normalized for GAPDH using ImageJ 1.44p software and values were given as relative units (RU). All the experiments were performed in triplicate.

### 2.9. Analysis of DNA Distribution and Spindle Configuration of In Vivo Matured MII Oocytes

In vivo matured MII oocytes were fixed for immunofluorescence and labelled by mouse anti-α-tubulin (T9026, Sigma Aldrich, 1:200) primary antibody overnight at 4 °C and secondary goat anti mouse- antibody conjugated with Alexa 594 (A90-137D4, Bethyl Laboratories Inc., 1:500) for 1 h at room temperature. Chromatin staining was performed by 5 μg/mL Hoechst 33342 (Sigma-Aldrich) for 5 min at room temperature. In negative control oocytes, the primary antibody was omitted. Oocytes were mounted on slides and analysed under epifluorescence microscope at 100× magnification.

### 2.10. Statistical Analysis

All data are presented as mean ± SEM. Statistical analysis was assessed by *t*-test. Analyses were performed using the SigmaStat software (Jandel Scientific Corporation, San Rafael, CA, USA). A *p*-value < 0.05 was considered statistically significant.

## 3. Results

### 3.1. Analysis of PCOS Phenotype in DHEA Mice

**Figure 1:**
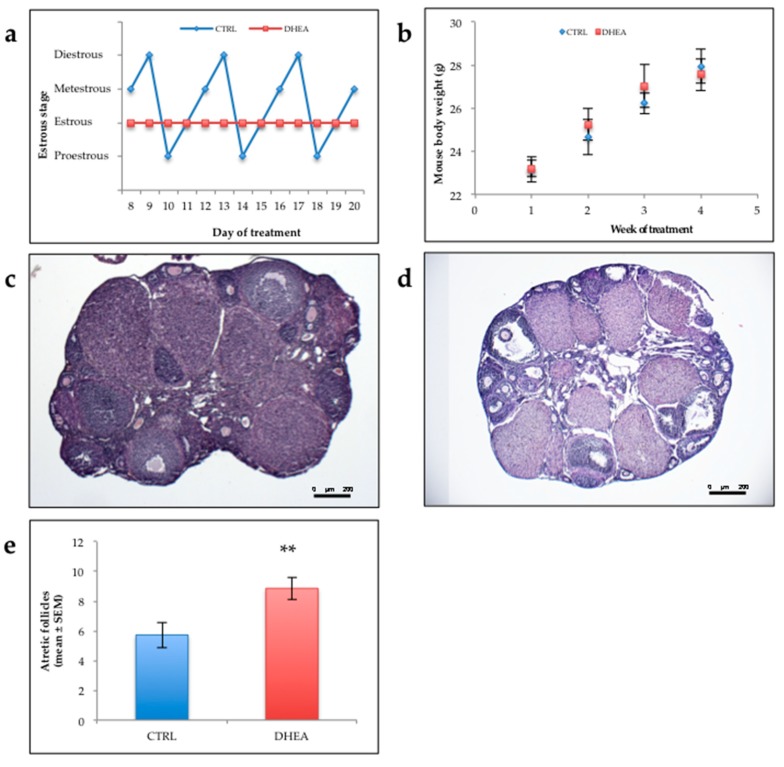
Estrous cycle, body weight, ovarian morphology in the mice. (**a**) Representative estrous cycle of one mouse from control and dehydroepiandrosterone (DHEA) group. (**b**) Body weight. Ten mice per experimental group were employed. (**c**,**d**) representative Haematoxylin and Eosin (H&E) staining of ovarian sections of CTRL (**c**) and DHEA (**d**) mice. (**e**) Atretic follicle. Three mice per experimental group were employed. Experiments were done in triplicate. **, *p* < 0.001, *t*-test.

Most mice of control group (80%) showed normal estrous cyclicity, while all the mice of DHEA group displayed abnormal estrous cycle. Representative cyclicities of mice in the two groups are showed in Figure 1a. No differences were observed in the weight of mice of the two groups as shown in Figure 1b.

Histologic examination of DHEA ovaries showed a looser appearance of the medullary and cortical stroma, respect to control ovaries (Figure 1c,d). While the latter showed the general histological organization of the mouse ovary—with an outer compact cortex rich of developing ovarian follicles, corpora lutea and atretic follicles interspersed in stromal elements and interstitial gland cells—the former were characterized by a loose aspect. More in detail, from a wide medullar stroma, rich of blood vessels embedded in a loose collagenous matrix, numerous sepiments protruded in the cortex to partially detach ovarian follicles and corpora lutea. The population of primordial, primary, secondary and antral follicles seemed normal in density and morphology. However, evident corpora lutea were seen, occasionally infiltrated by blood cells. Cystic-like structures with luteinized theca cells were occasionally found. Numerous atretic follicles, from all developmental stages but more from antral follicles, were also observed. Analysis of the follicle population in DHEA mice revealed that DHEA administration induced a highly significant in atretic follicles (Figure 1e) with respect to controls.

**Figure 2:**
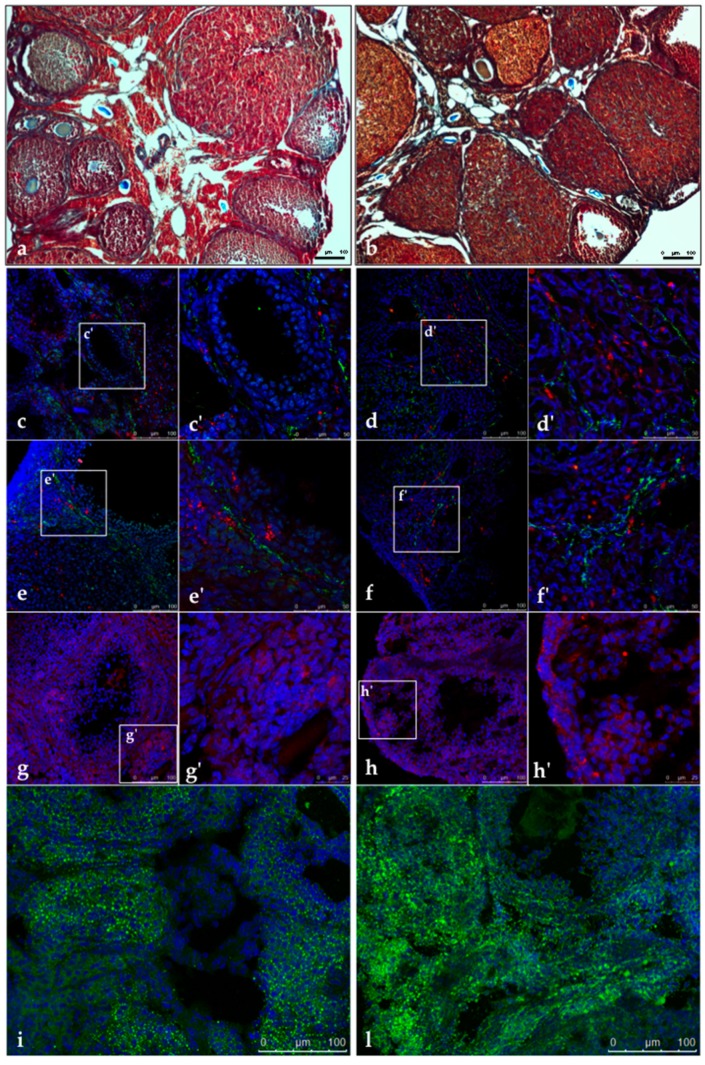
Representative images of trichrome staining in CTRL (**a**) and DHEA (**b**) mice. (**c**–**f**) Immunolocalization of Von Willebrand Factor (vWF) (green) and alpha smooth muscle actin (α-SMA) (red) in control (**c**,**c’** and **e**,**e’**) and DHEA (**d**,**d’** and **f**,**f’**) ovarian sections. (**g**–**h**) Immunolocalization of 17 beta-hydroxysteroid dehydrogenase type 4 (17β-HSD4) (red) in control (**g**) and DHEA (**h**) ovarian sections. Staining of lipid droplets by BODIPY 493/503 (green) in control (**i**) and DHEA (**l**) ovarian section. (**c**–**l**) DNA is stained by DAPI (blue). Three mice per experimental group were employed. Experiments were done in triplicate.

As shown in Figure 2a,b, the microscopic evaluation of DHEA ovaries subjected to trichrome stain evidenced a fibrotic aspect of ovarian cortex. In particular, the follicular wall of secondary and antral follicles showed a concentric and network-like collagen distribution, more intense than in controls. A thick and intense staining of collagen was detected also in medium-sized corpora lutea. As seen by H&E staining, blood cells infiltration was diffusely present in corpora lutea of DHEA ovaries.

The simultaneous staining for vWF, an endothelial cell marker (green stain) and α-SMA, a perycite marker (red stain) was used to identify the ovarian blood vessels. In both control and DHEA ovaries, vWF did not co-localize α-SMA (Figure 2c–f). The endothelial network was localized in the thecal layers of the ovarian follicles, and particularly in the antral follicles in both groups. In DHEA ovaries, endothelial cells were diffusely found located into corpora lutea, strictly associated to luteal cell as in atretic follicles. Groups of pericytes were spotted in the follicular and luteal walls. A diffuse staining was observed in the ovarian stroma, especially in DHEA ovaries. Overall, both vWF and α-SMA were more expressed in DHEA ovaries.

BODIPY staining after DHEA administration was stronger than in controls (Figure 2i,l). While in controls lipid droplets were visible as punctiform spots in the stroma, follicles and corpora lutea, DHEA ovaries showed the more abundant presence of larger lipid droplets. The anti 17β-HSD4 antibody immunostained strongly DHEA ovaries, with respect to controls (Figure 2g,h). The latter presented a diffuse and light staining in the ovarian stroma, more evident in the thecal layers of antral follicles and into the corpora lutea. DHEA ovaries showed an intense staining in the ovarian surface epithelium.

Finally, we confirmed the establishment of a condition of oxidative stress in DHEA mouse ovaries by evaluating lipid peroxidation [34]. As shown in Supplementary Figure S1, DHEA administration induced a significant increase in 4-HNE immunostaining.

### 3.2. DHEA Induced Ovulatory Dysfunction during Superovulation and Negatively Influences Oocyte Quality

**Figure 3:**
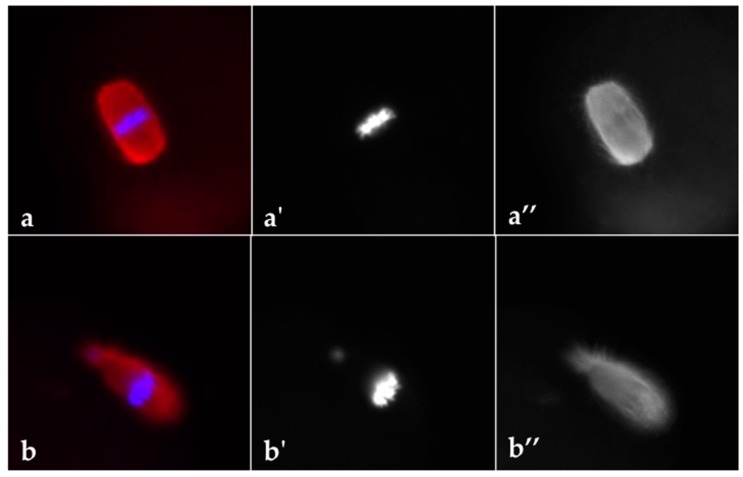
Representative images of MII plate observed in oocytes from control (**a–a’’**) and DHEA (**b–b’’**) mice following induction of ovulation. Spindle is stained by α-tubulin (red) and chromosomes are stained by Hoechst 33342 (blue). Five mice per experimental group were employed. Experiments were done in triplicate.

As reported in Table 1, DHEA administration negatively affected the mean number of ovulated oocytes per mouse after ovarian stimulation with gonadotropins (Table 1). Since normal metaphase configuration is crucial to oocyte quality, we analysed the effect of DHEA administration on spindle and chromosome organization in MII oocytes obtained following superovulation. The MII plate was classified as ‘normal’ when microtubules formed two opposite poles in association with a normal chromosomal distribution or ‘aberrant’ if microtubule structures displayed reduced dimensions of the spindle or lost normal poles or if disorganized microtubule patterns were observed in association with scattered, decondensed or disorganized chromosomes [35] (Figure 3). According to this classification, our data showed that percentage of oocytes with normal MII plate decreased about 40% in oocytes of DHEA group compared to control (Table 1).

### 3.3. Glycative Stress is Observed in the Ovary of DHEA Mice

**Figure 4:**
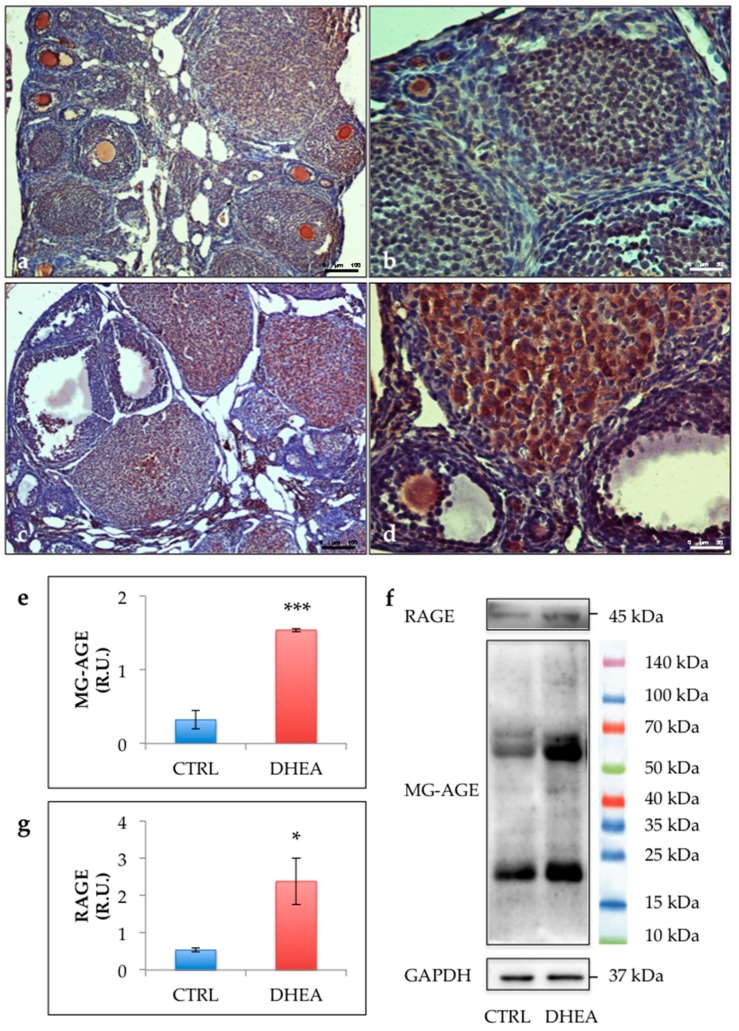
Representative images of immunolocalization of methylglyoxal (MG)-advanced glycation end-product (AGE) in control (**a**,**b**) and DHEA (**c**,**d**) ovaries. Western blot analysis of MG-AGE (**e**) and receptor for AGE (RAGE) (**g**) and representative images (**f**). Data are presented as means ± SEM of densitometric analysis of immunoreactive bands normalized to internal reference protein (glyceraldehyde-3-phosphate dehydrogenase, GAPDH). Three mice per experimental group were employed. Experiments were done in triplicate.*, *p* < 0.05; ***, *p* < 0.001, *t*-test.

**Figure 5:**
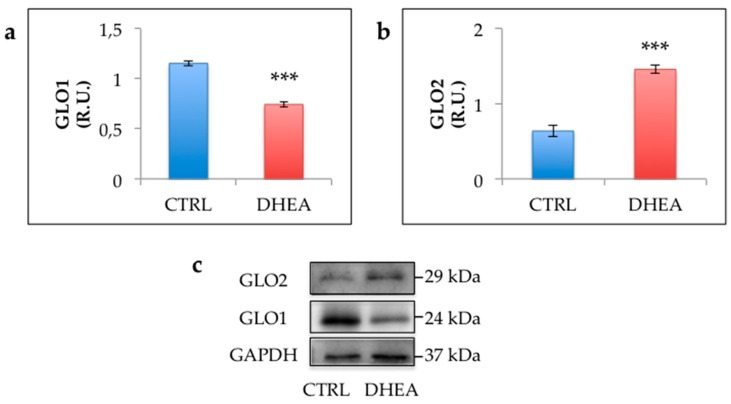
Western blot analysis of glyoxalase 1 (GLO1) (**a**) and GLO2 (**b**) and representative images of immunoreactive bands (**c**)**.** Data are presented as means ± SEM of densitometric analysis of immunoreactive bands normalized to internal reference protein (glyceraldehyde-3-phosphate dehydrogenase, GAPDH). Three mice per experimental group were employed. Experiments were done in triplicate. ***, *p* < 0.001, *t*-test.

The immunohistochemical analysis revealed an increased staining of MG-AGE in the ovaries of DHEA mice. Results obtained in control ovaries revealed low immunoreactivity in granulosa cells from primary, preantral and antral follicles, as well as in cumulus cells, oocytes and vessels. Nevertheless, low levels of MG-AGE staining could be observed in stromal cells. By contrast, sections of DHEA ovaries revealed an intermediate MG-AGE staining in oocytes and an intensive immunoreactivity in luteal cells, vessels and stromal cells (Figure 4a–d). This observation was confirmed by the comparison of the immunoreactive bands in DHEA and control groups (Figure 4e). MG-AGE accumulation was further supported by enhanced protein expression of RAGEs in the ovaries of DHEA mice (Figure 4f). To investigate the efficiency of the MG detoxification system, we assessed protein expression of GLO1 and GLO2. GLO1 protein expression was significantly reduced in ovaries from DHEA mice when compared with controls, whereas protein levels of GLO2 were increased (Figure 5).

### 3.4. SIRT1 Functional Network Regulating Mitochondrial Physiology Is Disrupted in DHEA Ovaries

**Figure 6:**
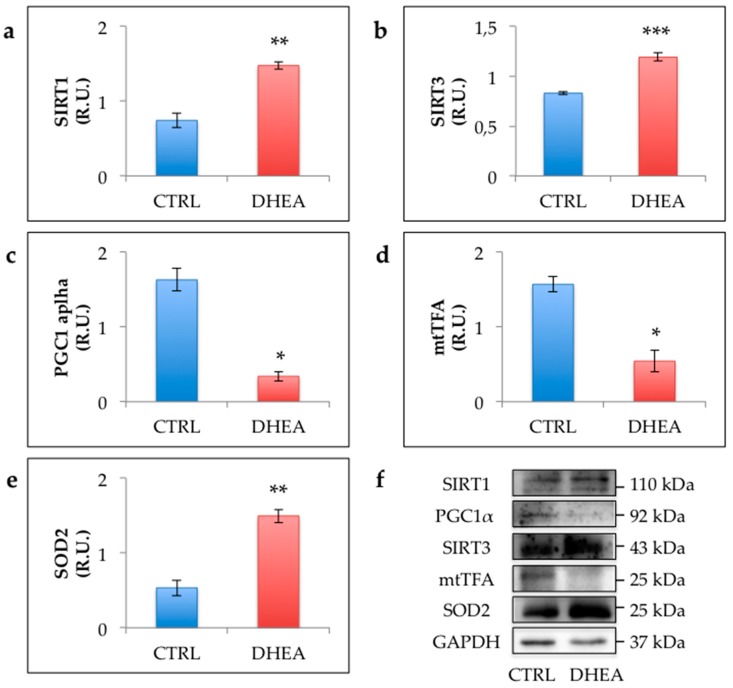
Western blot analysis of Sirtuin 1 (SIRT1) (**a**), SIRT3 (**b**), peroxisome proliferator-activated receptor gamma co-activator 1 alpha (PGC1α) (**c**), mitochondrial transcriptional factor A (mtTFA) (**d**) and superoxide dismutase 2 (SOD2) (**e**) and representative images of immunoreactive bands (**f**). Data are presented as means ± SEM of densitometric analysis of immunoreactive bands normalized to internal reference protein (glyceraldehyde-3-phosphate dehydrogenase, GAPDH). Three mice per experimental group were employed. Experiments were done in triplicate. *, *p* < 0.05, **, *p* < 0.01, ***, *p* < 0.001, *t*-test.

**Figure 7:**
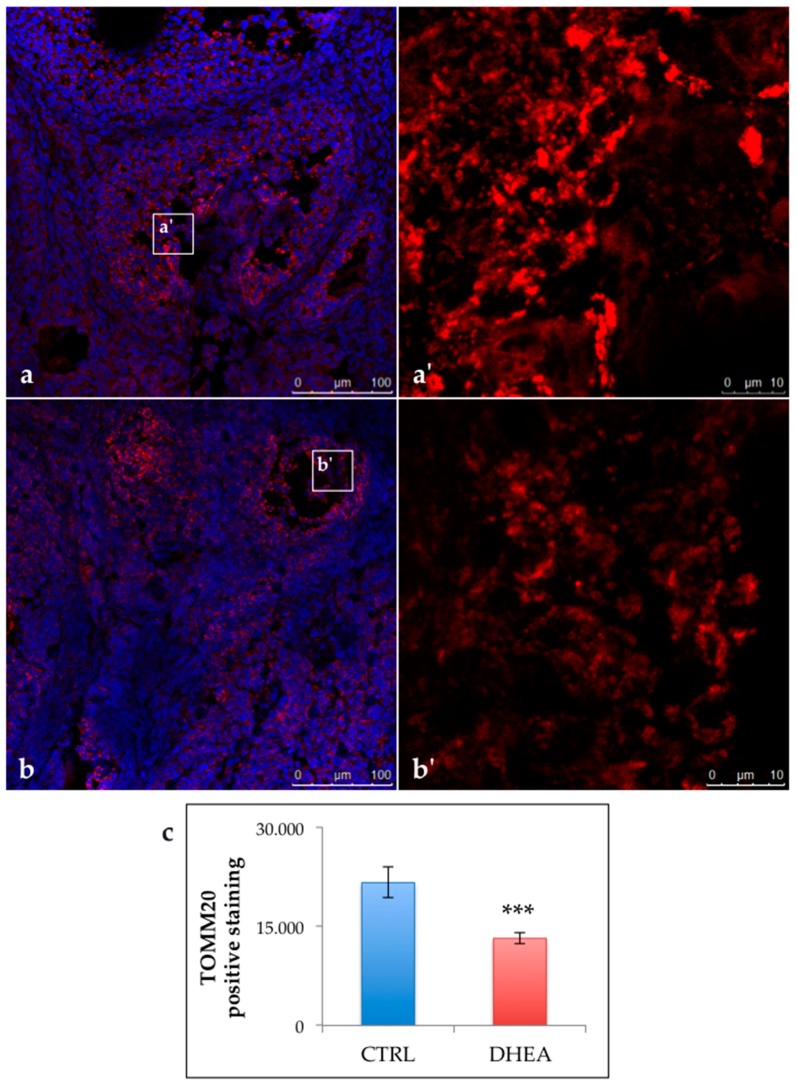
Immunolocalization and quantification of translocase of outer mitochondrial membrane 20 (TOMM20) positive staining (red). DNA is stained by DAPI (blue). Representative images of TOMM20 in CTRL (**a**,**a’**) and DHEA (**b**,**b’**) mice. Quantification of TOMM20 positive staining in the experimental groups (**c**). Data are presented as means ± SEM of mean pixel intensity of red fluorescence. Three mice per experimental group were employed. Experiments were done in triplicate. ***, *p* < 0.001, *t*-test.

To investigate whether PCOS induced by DHEA administration is associated with deregulation of SIRT1 functional network regulating mitochondrial physiology, we compared the levels of SIRT1 and proteins of SIRT1 mitochondrial network in control and DHEA mice. Data reported in Figure 6 revealed a significant increase of SIRT1 in DHEA mice. Moreover, we detected an upregulation of mitochondrial proteins SIRT3 and SOD2. Nevertheless, we observed a lower amount of PGC1α, the main regulator of mitochondrial biogenesis and function, and mtTFA, which reflects the rate of mtDNA transcription and mtDNA content. Confocal analysis of TOMM20 ovarian staining showed a reduced expression of the mitochondrial transporter in DHEA ovaries (Figure 7).

### 3.5. AMPK and Autophagy Increase in the Ovary of DHEA Mice

**Figure 8:**
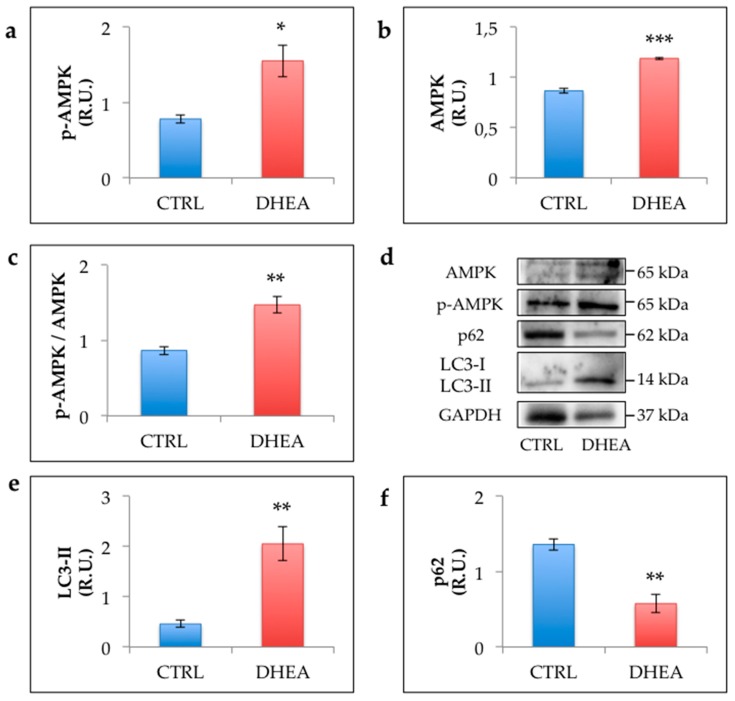
Western blot analysis of p-AMP-activated protein kinase (AMPK) (**a**), AMPK (**b**), p-AMPK/AMPK ratio (**c**), microtubule-associated protein light chain 3 (LC3)-II (**e**) and p62 (**f**) and representative images of immunoreactive bands (**d**). Data are presented as means ± SEM of densitometric analysis of immunoreactive bands normalized to internal reference protein (glyceraldehyde-3-phosphate dehydrogenase, GAPDH). Three mice per experimental group were employed. Experiments were done in triplicate. *, *p* < 0.05, **, *p* < 0.01, ***, *p* < 0.001, *t*-test.

Given the cooperation of SIRT1 with AMPK in regulating cell survival, we explored whether in PCOS mouse ovaries AMPK activation was associated with increased autophagy. As shown in Figure 8, we detected a significant increase of AMPK in DHEA mice. Moreover, we detected an upregulation of its activated form (phospho-AMPK) and an increased ratio of phospho-AMPK in comparison to total AMPK in DHEA mice confirming the recruitment of this enzyme. We also observed that DHEA ovaries exhibited higher levels of LC3-II and lower levels of P62 compared with control as an evidence of increased autophagy [36,37].

## 4. Discussion

Recently, it has emerged that overload of AGEs is a key factor in ovarian dysfunctions and reduced fertility associated with PCOS. Nevertheless, the specific role of MG, known as the most powerful AGE precursor involved in the pathogenesis of type 2 diabetes and several other age-related chronic inflammatory diseases, has been poorly investigated [38,39]. In our previous study we demonstrated that mice receiving oral MG administration for about one month presented early signs of the hyperandrogenic status associated with PCOS [17]. Moreover, Lin et al. [40] demonstrated that dietary supplementation with MG-BSA (bovine serum albumin) generated phenotypes similar to those observed in the PCOS rat model induced by DHEA. In the present work, we showed for the first time that MG-dependent glycative stress participates in the ovarian PCOS phenotype. Moreover, from our results it appears that this condition is associated with changes of SIRT1 functional network regulating mitochondrial physiology and cell survival.

Numerous rodent models have been established to study the mechanisms and possible therapies for PCOS [41]. In our study, we relied on a well-established DHEA-induced PCOS mouse model [20,41,42] and expanded the characterization of the ovarian microenvironment in DHEA mice. As expected, we detected anovulation in association with an increased number of atretic antral follicles [43]. In accordance with Huang et al. [44], DHEA mice employed in this study presented reduced ovulation rate and oocyte quality, in terms of altered meiotic spindle and chromosome configuration, following superovulation. This result is consistent with the hypothesis that alterations in oocyte competence underlie subfertility in many women with PCOS and confirms that hyperandrogenism is a main factor in this PCOS phenotype [45].

Further, we observed that DHEA ovaries were characterized by altered collagen deposition revealing the increased fibrotic tissue typically observed in the interstitial area of ovaries from PCOS patients [46]. We also provided evidence for increased vascularization in DHEA ovaries, which can be considered an effect of deregulation of ovarian angiogenesis contributing to abnormal follicular development in PCOS patients [47]. In DHEA mice it can be also observed a significant intraovarian accumulation of lipid droplets, which is consistent with altered fatty acid composition recently found in the follicular fluid of PCOS patients [48]. Intraovarian dyslipidemia may account for PCOS associated changes in follicle metabolism and for reduced oocyte competence in many PCOS patients [48]. Moreover, exposure of cumulus oocyte complexes to high lipid concentration is known to negatively influence oocyte maturation [49]. Finally, altered steroidogenesis at ovarian level is evidenced by increased expression of the isoform IV of the androgenic enzyme 17β-HSD in granulosa cells of DHEA ovaries, a finding that highlights the need for further investigation in PCOS women [50,51,52].

Different approaches have led us to demonstrate that MG-dependent glycative stress participates in the ovarian PCOS phenotype. Although MG levels have not been monitored, the finding of increased MG-AGE deposition represents an evidence of disrupted balance in the process of detoxification/formation of MG in the ovary of DHEA mice. The potential role of MG in ovarian dysfunctions has been well established in previous studies from our research group [22,23,24]. Based on in vitro studies, exposure to supraphysiological MG concentrations impairs oocyte meiosis and decreases inner mitochondrial redox potential and distribution [53]. Investigation on the regulation of glyoxalases has led us to demonstrate that upregulation of GLO1 and GLO2 expression in response to MG insult is dependent on SIRT1 activity in mouse oocytes. Similarly, oral administration of MG resulted in the up-expression of ovarian SIRT1 functional network and GLO1, as components of an adaptive response capable to counteract MG-AGE accumulation [17]. By contrast, here we observed that DHEA administration was responsible for increased intraovarian MG-AGE levels in association with enhanced expression of RAGEs and deregulation of the glyoxalase system, represented by reduced expression of GLO1, a well-known hallmark of glycative stress, and increased GLO2 level. MG accumulation in DHEA ovaries may derive from both altered glucose metabolism [41] and oxidative stress [10]. Here, oxidative stress has been evidenced by increased lipid peroxidation in DHEA ovaries in accordance with data previously reported in DHEA Balb/C mice [54]. MG increases as a result of reduced GLO1 protein, which is likely to be associated with reduced GLO1 activity, as it occurs in aged mouse ovaries [22]. Indeed, oxidative damage, by altering GSH availability, may negatively affect GLO1 activity. Furthermore, glycative stress may account for increased fibrosis and altered vascularization here observed in DHEA ovaries.

Recently, the interest in the involvement of SIRT1 in PCOS development and progression has been increasing [19]. Here we found that DHEA mice exhibited enhanced ovarian expression of SIRT1. Indeed, increased expression of sirtuins is considered an adaptive response to mild oxidative stress, whereas severe oxidant conditions determined sirtuin degradation [55]. The recruitment of SIRT1 in DHEA ovarian response to prooxidant conditions is supported by the rise in protein levels of SIRT3 and SOD2, which are mitochondrial elements of SIRT1 functional network involved in antioxidant response. Nevertheless, this machinery is not able to prevent mitochondrial failure evidenced by the decrease of PGC1α, mtTFA and TOMM20. This clearly indicated that a decrease in mitochondria number occurred in PCOS. Indeed, PGC1α is the major regulator of mitochondrial biogenesis and function, whereas mtTFA is mitochondrial transcription factor whose levels directly reflect the levels of mtDNA content, thus being considered a marker of mitochondrial number [25,26]. In strong accordance with this finding, the reduction in mitochondrial number was further confirmed by semi-quantitative immunofluorescence-based analysis for TOMM20, a protein in the outer mitochondrial membrane, which is also considered a marker of mitochondrial mass [56]. Therefore, a decrease of resident mitochondrial proteins would be expected in case of reduced mitochondrial pool. Conversely, we found that SOD2 and SIRT3, both located in mitochondria and strictly involved in mitochondrial antioxidant defense [57,58], were up-regulated in DHEA mice, as compared to control mice. This counterintuitive trend clearly suggests that PCOS altered the redox milieu within ovarian mitochondria, as indicated by the marked activation of the SIRT3/SOD2-driven antioxidant response. Our results support the involvement of mitochondria in this disease and represent the first evidence of mitochondria deregulation at ovarian level [59]. Further investigation will be required to clarify the relationship between ovarian mitochondrial changes here described in DHEA mice and energy production.

Based on the recent finding of increased autophagy in PCOS ovarian cells [28,29], we investigated the presence of this PCOS phenotype in DHEA mice. As recently reviewed by Yoshii and Mitzushima [37], the increase in LC3-II we found in this study represents an evidence that DHEA-induced PCOS is characterized by augmented autophagic flux. This was further confirmed by the data related to p62, which decreases when autophagy is induced [36]. It is important to remark that SIRT1 regulates autophagy machinery through multiple mechanisms, including direct deacetylation of autophagy-related proteins such as LC3 [60], or via the activation of AMP-activated kinase (AMPK), another important energy sensor [27]. In the present study, we found that up-regulation of SIRT1 in DHEA ovaries is associated with increased AMPK activation and the presence of autophagy markers, thus indicating that a SIRT1/AMPK axis modulates autophagy in PCOS ovaries.

Overall, present findings represent an important contribution to the characterization of biochemical markers of DHEA mouse model and to elucidation of new molecular mechanisms underlying PCOS development or progression at ovarian level. In particular, our results demonstrate the role of MG in the glycative stress detected in PCOS and highlight the need for further investigation of the implication of SIRT1, mitochondria and autophagy in the pathogenesis of PCOS. Approaches aimed to reduce MG burden can potentially form the basis for new treatment strategies for ameliorating PCOS fertility potential.

## Figures and Tables

**Table 1:** Effect of DHEA on ovulatory function and oocyte quality.

	CTRL (Mean ± SEM)	DHEA (Mean ± SEM)
No. of oocytes from per mouse	17.4 ± 1.0	10.8 ± 1.0 **
Percentage of MII oocytes	92.1 ± 1.2%	86.4 ± 3.9%
Percentage of MII oocytes with normal metaphase plate	77.2 ± 4.0%	44.0 ± 7.5% **
Percentage of degenerated oocytes	8.0 ± 1.2%	13.6 ± 3.9%

**, *p* < 0.01, *t*-test.

## Supplementary Materials

The following are available online at https://www.mdpi.com/2073-4409/9/1/209/s1, Figure S1: Immunolocalization and quantification of 4-HNE positive staining in ovaries from control and DHEA mice.
