# Supplementary material for: Methylglyoxal-Dependent Glycative Stress and Deregulation of SIRT1 Functional Network in the Ovary of PCOS Mice
Source: Cells. 2020 Jan 14;9(1):209. doi: 10.3390/cells9010209 (PMC7017084; doi:10.3390/cells9010209)
Supplement: Supplementary file 1 [file cells-09-00209-s001.pdf]

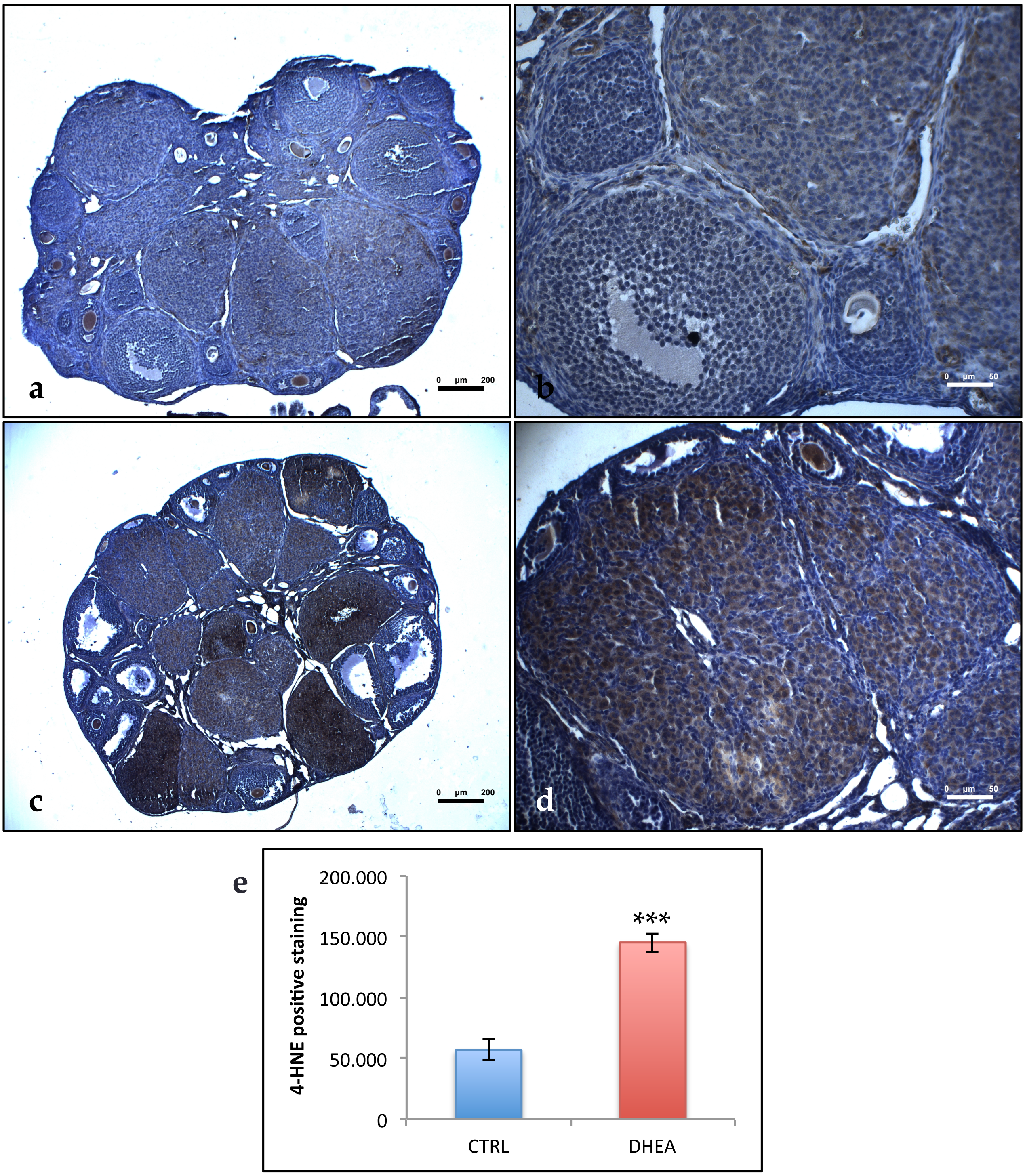

Supplementary Figure 1. Immunolocalization and quantification of 4-HNE positive staining. Representative images of 4-HNE in CTRL (a, b) and DHEA (c, d) mice. Quantification of 4-HNE positive staining in the experimental groups (e). Data are presented as means ± SEM of mean pixel intensity of 4-HNE signals. Three mice per experimental group were employed. Experiments were done in triplicate. ***P<0.001, t-test.
